# A Four-Step Method for Optimising the Normal Water Level of Reservoirs Based on a Mathematical Programming Model—A Case Study for the Songyuan Backwater Dam in Jilin Province, China

**DOI:** 10.3390/ijerph8041049

**Published:** 2011-04-07

**Authors:** Shijun Sun, Xiaofei Yan, Peng Cui, Jiang Feng

**Affiliations:** College of Urban and Environmental Sciences, Northeast Normal University, Changchun 130024, China; E-Mails: sunsj74@126.com (S.S.); xf-yan@126.com (X.Y.); cuip006@126.com (P.C.)

**Keywords:** normal water level, dam construction, mathematical modelling, system analysis, significance test, correlation analysis, principal component analysis, sensitivity analysis

## Abstract

Determination of the optimal normal water level of reservoirs (RNWL) was investigated, incorporating environmental ecology as a primary consideration. RNWL constitutes a relatively significant eigenvalue of any water conservancy project. In the present study, a four-step method based on a mathematical programming model and suitable for RNWL decision making was developed and applied to the water conservancy project of the Songyuan backwater dam in China. System analysis, correlation analysis, significance testing, principal component analysis, sensitivity analysis, and system optimisation theory are used in the solution process. In this study, various factors that impact the economic viability, engineering characteristics, environmental and urban ecology are considered for holistic optimisation. The study shows that the proposed four-step method may provide a feasible quantitative form of support for RNWL decision making.

## 1. Introduction

A balanced ecological-economic water system is crucial for social development. In fact, the construction of dams has contributed to rapid human development by providing reliable sources of drinking water, crop irrigation, hydropower, recreation, navigation, income, in addition to a number of other important benefits [1]. However, dam construction also has negative ecological consequences on the structure, processes, and functioning of ecosystems [2], including deforestation, loss of fauna and flora species, and the destruction of historical remains [3].

Determining the optimal normal water level of a reservoir (RNWL) is considered to be the most significant eigenvalue of a dam construction project. This parameter may have direct impacts on the scope of project and environmental ecology, as well as other characteristics, such as capacity effectiveness, flow regulation, and comprehensive utilisation benefit. Furthermore, reservoir land and recreational values are dependent on water levels remaining at useable levels. Thus, comprehensive considerations of economic, engineering and eco-environmental parameters by quantitative means are required to optimise RNWL.

Several methods for optimising RNWL have been increasingly used over the last few decades. For example, Lu [4] proposed a new method for selecting RNWL by using grey layers analysis, in which the weighted coefficient of each index was determined by means of excavating, observing and including the depth information in a formative process of the indices. Zhang and Qu [5] provided a new method for optimisation of RNWL based on ambiguous relation analysis, which proved reliable in practical applications. Zeng *et al.* [6] developed a multi-objective decision-making model by using grey correlation analysis for the selection of RNWL of the Three Gorges reservoir. For the comprehensive evaluation of RNWL schemes, Jin *et al.* [7] proposed an objective weight method based on the projection pursuit according to the sample series of water criterion and improved analytic hierarchy process based on an accelerating genetic algorithm. Xie and Qian [8] used the grey fuzzy comprehensive assessment method to select a RNWL to quantify qualitative indices by using the fuzzy number and the relationship of the indices being considered. Hou [9] presented the application of the multi-principle appraisal method in the selection of RNWL, using a real hydropower plant as example. However, to date most existing studies have focused on selecting RNWL through the comprehensive evaluation of RNWL schemes, which while reasonable, in fact has a low level of accuracy. Although mathematical programming models are very useful for obtaining more accurate and reliable RNWL for the whole system, few studies have systematically optimised RNWL through mathematical modelling, despite the availability of quantitative information. The reason for this gap is because it is difficult to adequately describe all the factors that have an impact when using mathematical language.

Therefore, the objective of this study was to develop a new method that is suitable for RNWL decision making, which is based on a mathematical programming model that incorporates available quantitative information. The four-step method combines the technologies of system analysis, correlation analysis, significance testing, principal component analysis, sensitivity analysis and the theory of system optimisation, and was applied to the Songyuan backwater dam water conservancy project in China.

## 2. Methodology

**Figure 1.**
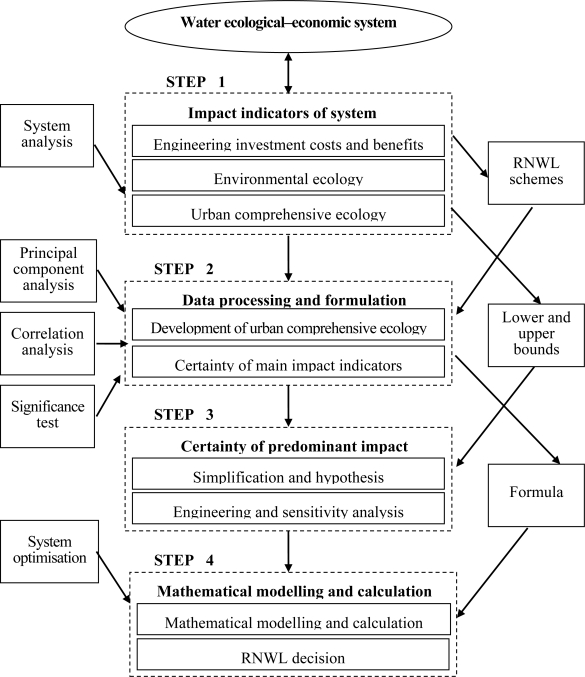
Framework of the new method for RNWL decision making.

An ecological-economic water system is usually very complex, with the RNWL decision making process requiring environmental, economic, engineering and social considerations. However, it is unnecessary for researchers to incorporate all the factors that impact the system in a single mathematical programming model. Each decision-making process starts with problem recognition, followed by information search, problem analysis, alternative evaluation, and finally the decision [10]. The four-step method requires the following phases, shown in Figure 1.

Step 1: In our study, factors related to RNWL that impact the ecological–economic water system are best considered in as comprehensive terms as possible. Hence, we proposed that these factors are divided into three categories based on the technology of system analysis: engineering investment cost and benefits, environmental ecology, and urban comprehensive ecology. System analysis in this paper mainly includes the following steps:

1. Project and environment impact analysis
2. Raw classification. In this step, brainstorming, the consultation of experts, analytical hierarchy process, and other system analytical methods may be used based on the actual situation.
3. Evaluation of raw classification. Reasonable classification is more convenient for mathematical modelling, although it is the first step of the methodology. In this section, the model framework of RNWL optimisation must be formed to evaluate the classification.
4. Classification adjustment.

According to the results of the previous step, the classification should be adjusted until it fits the model framework for the modeller. Table 1 shows the recommended classification in this study [4–9,11], based on experience. In general, most dam construction evaluations include the impact indictors shown in Table 1. In an actual real-life situation, researchers may select additional or alternative indictors. Meanwhile, the bounds of RNWL require identification and selection of a number of schemes within this interval. The purpose of selecting a number of schemes is to study the proximity and significance of the relationship between RNWL and the impact indictors that are grouped into engineering investment costs and benefits and environmental ecology. The data for the indicators of RNWL schemes should be assimilated for subsequent analysis in Step 2.

Step 2: Manages the years of data from the impact indicators that are grouped into urban comprehensive ecology through principal component analysis [12], in order to develop the formula for mathematical modelling in Step 4. Furthermore, the main impact indicators that belong to engineering investment costs and benefits and environmental ecology in RNWL decision making, should be defined through correlation analysis and significance testing. The main purpose of correlation analysis is to study the closeness of the relationship between impact indictors and RNWL. Meanwhile, the significant relationship is determined through significance testing [13]. Assuming significance (hereinafter “sig.”) equals 0.05 [14], the critical value of the related coefficient (hereinafter “R”) is obtained [15]. First, we compare the sig. of the impact indictors with 0.05. If the sig. >0.05, the indictors are rejected, otherwise R of the remaining impact indictors must be compared with the critical value, and we could select the main indictors based on R ≥ critical value.

Step 3: The objective of RNWL decision making must be identified, and according to the characteristics of a real-world problem, a reasonable simplification and associated assumptions should be put forward. While the upper and lower bounds of RNWL are identified in Step 1, engineering and sensitivity analysis of the main impact indicators are used to determine the dominant impact indictors that should be incorporated in the mathematical programming model.

Step 4: Mathematical modelling and calculation. The predominant impact indictors determined in Step 3 are described as model objectives and constraints by using mathematical language, and the mathematical programming model for RNWL decision making is developed.

## 3. Application

### 3.1. Analysis of the Study System

As a case study the methodology described above was applied to a backwater dam project on the second Songhua River of Songyuan City. The urban area of Songyuan City is located between the Qianfu Bridge and the Longhua Bridge, with water level of dry seasons ranging from 129.2 m to 129.5 m and water surface occupancy of both sides of the dykes reaching only 20%. The purpose of the backwater dam is to elevate the water level of the urban city zone in dry seasons to improve the water environment and meet the urban landscape planning requirements. Specifically, the river inflow is planned to be entirely discharged to downstream with no closure. The backwater dam is about 32 km away from upstream Hatta Mountain station and 4.5 km away from downstream Fuyu hydrological station which is programmed to be moved away. The recommended scheme obtained by the conventional method is 131.5 m. Based on the analysis and feasibility study report of Songyuan ecological-economic water system, the considered impact indicators and classification are shown in Table 2. Meanwhile, the identified bounds of RNWL are 131.4 m and 132.1 m with [131.4, 132.1] m as the general constant interval and four schemes are selected as follows: 131.4 m, 131.6 m, 131.8 m and 132.1 m.

### 3.2. Data Processing and Formulation

Data for the impact indicators grouped into urban comprehensive ecology from 2002 to 2009 were collected. In addition, data for nine impact indictors belonging to engineering investment costs and benefits and environmental ecology were collected.

In this procedure, correlation analysis and significance testing in SPSS [13] is used to define the main impact indictors (Table 2). On the other hand, the formula of urban comprehensive ecology is developed through principal component analysis [12], as follows:

(1) $$\text{VEI}=\omega_{\text{SD}}\times V_{\text{SD}}+\omega_{\text{EE}}\times V_{\text{EE}}+\omega_{\text{ED}}\times V_{\text{ED}}$$

where *VEI* represents the ecosystem colligate index of Songyuan city; *ω_SD_* denotes the weights of social development indexes; *V_SD_* is the social development indexes; *ω_EE_* is the weights of eco-environment development indexes; *V_EE_* represents eco-environment development indexes; *ω_ED_* denotes the weights of economic development indexes; and *V_ED_* is the economic development indexes, respectively. Furthermore, the weights of indexes in Equation 1 are obtained from the mean square error method, which is considered to be objective [12].

### 3.3. Certainty of Predominant Impact Indictors

The objective of RNWL decision making is to elevate the water level of the urban city zones in dry seasons, thus taking into consideration Songyuan city’s comprehensive ecological and environmental protection scheme. As a result, a series of simplification and assumptions are put forward:

1. The operational period of Songyuan backwater dam is 40 years;
2. 129.5 m is the minimum draw-down reservoir water level;
3. The dam cross section is trapezoidal;
4. Only the height of the dam changes under different RNWL in the interval [131.4, 132.1] m;
5. Sewage treatment plants will not operate until the reservoir meets RNWL levels.

Obviously, the three predominant indictors are: (1) area of land submergence, (2) engineering construction costs, and (3) water surface occupancy. In comparison, the remaining predominant impact indictors may be defined through using the engineering and sensitivity analysis as follows:

(1) Backwater length is in direct proportion to RNWL. If the backwater length of the upper bounds of RNWL is less than the distance between the dam site and Hatta Mountain station (*i.e.*, 32 km), changes in the RNWL interval [131.4, 132.1] m would not be sensitive to it.

In this scenario, the backwater length for 132.1 m is 23.15 km, which is below 32 km. Therefore, the backwater length is not a predominant impact indictor to this project.

(2) Variation of sand sediment volume is the same as backwater length. Based on the assumption and engineering analysis, the maximum sand sediment volume for 40 years is 917 × 10^4^ m^3^, and for the lower and upper bounds of RNWL this is 648.4322 × 10^4^ m^3^ and 1085.803 × 10^4^ m^3^, respectively [16].

Consequently, sand sediment volume appears to be the predominant impact indictor.

(3) The structure of reservoir water temperature is generally divided into hierarchical, transitional and mixed type. The sensitivity of RNWL changing at intervals of [131.4, 132.1] m to water temperature may be determined from the ratio of runoff and storage capacity (hereinafter “α”) [16]. As shown in Table 3, the values of α all exceed 20. In comparison, the sensitivity of RNWL changing at intervals of [131.4, 132.1] m when the water temperature structure is of mixed type with a characteristic uniform temperature distribution, there would be no significant heat exchange between the surface and the bottom (with basically the same horizontal and vertical temperatures), that is, water of low temperature would not be discharged and there would be negligible impact to the aquatic environment. Therefore, water temperature does not appear to be a predominant impact indictor.

(4) This project incorporates both positive and negative impacts on water quality, which are mainly focused on the quality of reservoir water during the period of operation. Hence, while the dilution capacity is enhanced by raising RNWL, the self-purification ability is reduced.

In such conditions, determining the sensitivity of changes in RNWL interval [131.4, 132.1] m to water quality is complicated, and may be regarded as a predominant impact indictor.

In total, five main impact indictors were found to be predominant: (1) area of land submergence, (2) engineering construction cost, (3) water surface occupancy, (4) sand sediment volume, and (5) water quality (Table 2).

### 3.4. Mathematical Modelling and Calculations

The minimum of the total engineering investment cost and eco-environmental impact is the optimisation criterion that expresses the efficiency of the system, when taking into account the comprehensive urban ecology of Songyuan City.

(2) $$\begin{array}{l} \text{MinE}=E_{c}+E_{f}\\ =L\times \left[\frac{1}{2}\times {\left(X_{\text{wl}}-H_{b}\right)}^{2}\times \left(\frac{1}{r_{1}}+\frac{1}{r_{2}}\right)+a\times \left(X_{\text{wl}}-H_{b}\right)\right]\times \left(C^{e}\times P^{e}+\sum_{i=1}^{6}C_{i}^{v}\times P_{i}^{v}\right)+\\ \left\{\left(\frac{1}{r_{1}}+\frac{1}{r_{2}}\right)\times {\left(X_{\text{wl}}-H_{b}\right)}^{2}+\left[2\times a+L\times \left(\frac{1+\sqrt{1+r_{1}^{2}}}{r_{1}}+\frac{1+\sqrt{1+r_{2}^{2}}}{r_{2}}\right)\right]\times \left(X_{\text{wl}}-H_{b}\right)+2\times a\times L\right\}\\ \times \sum_{j=1}^{4}C_{j}^{s}\times P_{j}^{s}+C_{a}^{f}\times A_{a}^{f}+C_{w}^{f}\times A_{w}^{f} \end{array}$$

where *X_wl_* is the decision variable representing the value of RNWL (m); *E* denotes the investment cost ($); *E_c_* is the mean cost of the overflow earth dam ($); *E_f_* stands for the cost of land submergence ($); *L* is the length of the overflow earth dam (m); *H_b_* is the elevation of the base of the dam (m); *r_1_* and *r_2_* are upstream and downstream slopes; a is top side length of the overflow earth dam (m); *C^e^*, *C_I_^V^* and *C_j_^s^* are the unit prices of earth and stone for filling and impervious materials ($/m^3^); *P^e^*, *P_i_^v^* and *P_j_^s^* are the proportion (%) of volume of substrate removal, earth and stone for filling and types of impervious material required for the overflow earth dam; *C_a_^f^* and *C_w_^f^* are the cost of per unit of inundated area of farmland and woodland ($/m^2^); *A_a_^f^* and *A_w_^f^* are the inundated area of farmland and woodland (m^2^), respectively.

As shown in Equation (2), which is considered objective, the predominant indictors of engineering quantity and the area of land submergence are described by mathematical language as the engineering quantity cost of the overflow earth dam and the compensation fee of land submergence, respectively.

The model constraints impose limits on the problem variable and include:

1. Urban landscape planning constraint:
  (3) $$R_{\text{min}}\leq 20.31X_{\text{wl}}+4.6\leq R_{\text{max}}$$
  where *R_min_* and *R_max_* represent the lower and upper bounds of water surface occupancy (%).
2. Maximal sediment volume constraint [16]:
  (4) $$\frac{40\times S\times \left(V_{\text{wl}}-V_{\text{rs}}\right)}{\rho \times \left[0.012\times W_{e}+0.0102\times \left(V_{\text{wl}}-V_{\text{rs}}\right)\right]}\leq V_{\text{rs}}$$
  where *S* denotes sediment storage (t/a); *V_wl_* is the mean corresponding capacity of RNWL (m^3^); *V_rs_* is dead storage (m^3^); *ρ* is dry density (t/m^3^); *W_e_* is the average water storage per year (m^3^/a).
3. Reservoir water quality constraint [16]:
  (5) $$\frac{W_{o}}{K_{h}\times V_{\text{wl}}}+\left(c_{h}-\frac{W_{o}}{K_{h}\times V_{\text{wl}}}\right)\times e^{\left(-K_{h}\times t\right)}\leq c_{0}$$
  where *W*_0_ is the rate of storage pollutants (g/s); *K_h_* is the intermediate variable (s^−1^); *c_h_* is the chemical oxygen demand (COD)of the water (mg/L); *t* is uniformly mixed time (s); *c_0_* is the average COD of the water for the years before the operation of the backwater dam (mg/L).
4. Urban comprehensive ecological index (UCEI) constraint [12]:
  (6) $$\omega_{\text{SD}}\times V_{\text{SD}}+\omega_{\text{EE}}\times V_{\text{EE}}+\omega_{\text{ED}}\times V_{\text{ED}}\geq 0.80$$
  where 0.80 is the lower bound of the city ecosystem colligate index, which indicates that the city represents a healthy urban ecosystem [11].

As shown in Equations (2)–(5), aside from the indictors of engineering quantity and area of land submergence, the other three predominant indictors (water surface occupancy, sand sediment volume, and water quality) are all described by mathematical language as the constraints imposed on the value of RNWL. These indicators are embodied in the constraints of urban landscape planning, maximal sediment volume, and reservoir water quality. Furthermore, consideration of the comprehensive ecology of the urban city of Songyuan embodies the constraint of the urban comprehensive ecological index, as shown in Equation (6).

## 4. Results and Discussion

Table 4 presents the results obtained from the four-step method, which was based on a mathematical programming model. From this model, the recommended RNWL of the Songyuan backwater dam is 131.40 m, with the two latter decimal points being reserved. The model indicates that the new solution for optimising RNWL may support the decision making process based on effective quantified indicators. Hence, determining the global optimum may be obtained through more comprehensive consideration of engineering economics, eco-environment protection, and urban comprehensive ecology. In comparison to the our ecological-economic modelling system results, the recommended RNWL for the dam based on the conventional method (less accurate) was 131.5 m, which was selected through evaluating 131.0 m, 131.5 m, 132.0 m and 132.5 m. Table 5 presents the results for 131.5 m from the mathematical programming model developed in the fourth step of this study.

As shown in Tables 4 and 5, the engineering quantity cost and compensation fee of land submergence based on the scheme of 131.40 m are both below that of the 131.5 m scheme. Comparison of the above results means that either the minimum of the total engineering investment cost or minimum of ecological environment impact must be selected as the optimisation criterion. Basically, scheme 131.40 m is better than 131.5 m for the whole system. Despite certain impact indictors being slightly inferior in the 131.40 m system (*i.e.*, UCEI, water surface occupancy and water quality) there is no fundamental difference in these parameters, all of which meet the requirements of this project under the criteria of the two schemes.

## 5. Conclusions

A new method based on four-step mathematical programming modelling for identifying the global optimal value for the feasible interval of RNWL in the ecological-economic water system has been introduced for water conservancy projects. This method systematically combines the technologies of system analysis, correlation analysis, significance testing, principal component analysis, sensitivity analysis and the theory of system optimisation.

Compared with existing methods, the four-step method may reduce certain uncertainties caused by human factors, in addition to providing a series of effective quantified indicators for the decision maker. Meanwhile, the optimisation results of Songyuan backwater dam indicate that the optimum RNWL obtained by using the new method are more accurate and reliable.

Although this study is only the first attempt to optimise RNWL through the development of mathematical modelling, the results suggest that this hybrid technique is effective, and may be applied to water conservancy projects under certain conditions. The mathematical model developed here may also be integrated with other methods to further enhance its data processing and evaluation capacity.

## Figures and Tables

**Table 1.** Consideration and classification of the potential impact indicators.

**Category**		**Potential impact indicators**
Engineering investment cost and benefits		Area of land submergence Engineering construction cost Power generation income Project immigrants Backwater length Special facilities Flood control project of reservoir downstream
Environmental ecology		Water surface occupancy Sediment condition Water quality Water temperature Groundwater quality Downstream flow
Urban comprehensive ecology	Social development indicators	Natural growth rate of population Population density The number of students at university or college per ten thousand people Life expectancy Urbanisation GDP proportion of investment in science and education Road area per capita Housing area per capita Hospital beds per ten thousand people Domestic water per capita Power consumption per capita Engel coefficient Insurance premiums per capita Unemployment rate Amount of book collection per ten thousand people Probability of occurrence of criminal cases
	Ecological and environmental development indicators	Green area per capita Air humidity Standard emission rates of industrial wastewater Annual emissions of industrial COD Annual average of inhalable particulate matter Industrial SO_2_ emissions Industrial soot emissions Industrial dust emissions Average noise of main roads of the city Noise environment quality Comprehensive utilisation of industrial solid waste GDP proportion of investment in environmental protection City river runoff depth
	Economic development indicators	GDP per capita GDP annual growth rate GDP proportion of tertiary industry Urban-rural income ratio Investment results City tourism revenue

**Table 2.** Indictors of Songyuan backwater dam.

**Category**	**Step1**	**Step 2**	**Step3**

	**Impact indicators**	**Main indicators**	**Predominant indictors**
Engineering investment costs and benefits	Area of land submergence	•	✓
	Engineering construction cost	•	✓
	Backwater length	•	
	Special facilities		
	Flood control project of reservoir downstream		
Environmental ecology	Water surface occupancy	•	✓
	Sand sediment volume	•	✓
	Water quality	•	✓
	Water temperature	•	

**Table 3.** Ratio of runoff and storage capacity.

**Average runoff (×10^8^ m^3^)**	**RNWL (m)**	**Total storage capacity (×10^8^ m^3^)**	**α**	**Type**	**Remarks**
103.57	131.4	0.3365112	307.77	mixed	If α < 10, hierarchical type;
	132.1	0.5060861	204.65	mixed	If α > 20, transitional type;
					If 10 < α < 20, mixed type.

**Table 4.** Optimisation results from the new method.

**RNWL (m)**	**Investment ($10^6^)**		**UCEI**	**Water surface occupancy (%)**	**Sediment volume (10^4^ m^3^)**	**Water quality (mg/L)**
	**Engineering quantity cost**	**Compensation fee**				
131.40	1.75	9.00	0.895	40	653.363	6.378

**Table 5.** Results of the conventional method.

**RNWL (m)**	**Investment ($10^6^)**		**UCEI**	**Water surface occupancy (%)**	**Sediment volume (10^4^ m^3^)**	**Water quality (mg/L)**
	**Engineering quantity cost**	**Compensation fee**				
131.5 m	1.85	10.34	0.896	42	709.421	6.003
